# Dynamic Changes of Gene Expression in Mouse Mural Trophectoderm Regulated by *Cdx2* During Implantation

**DOI:** 10.3389/fcell.2022.945241

**Published:** 2022-08-16

**Authors:** Daisuke Suzuki, Keisuke Sasaki, Soichiro Kumamoto, Keisuke Tanaka, Hidehiko Ogawa

**Affiliations:** ^1^ Department of Bioscience, Graduate School of Life Science, Tokyo University of Agriculture, Tokyo, Japan; ^2^ Bioresource Center, Graduate School of Medicine, Gunma University, Maebashi, Japan; ^3^ NODAI Genome Research Center, Tokyo University of Agriculture, Tokyo, Japan

**Keywords:** Cdx2, mural trophectoderm, implantation, transcriptome, blastocyst

## Abstract

Implantation of the blastocyst into the uterus is a specific and essential process for mammalian embryonic development. In mice, implantation is initiated from the mural trophectoderm of the blastocyst and the mTE controls implantation progression by acquiring the ability to attach and invade into the endometrium while differentiating into primary trophoblast giant cells. Nevertheless, it remains largely unclear when and how the mTE differentiates and acquires this ability during implantation. Here, by RNA sequencing analysis with the pre- and peri-implantation mTE, we show that the mTE undergoes stage-specific and dynamic changes of gene expression during implantation. We also reveal that the mTE begins down-regulating *Cdx2* and up-regulating differentiation marker genes during the peri-implantation stage. In addition, using trophectoderm (TE) -specific lentiviral vector-mediated gene transduction, we demonstrate that TE-specific *Cdx2* overexpression represses differentiation of the mTE into the primary trophoblast giant cells. Moreover, we reveal that TE-specific *Cdx2* overexpression also represses the up-regulation of cell adhesion- and migration-related genes, including *Slc6a14*, *Slc16a3*, *Itga7*, *Itgav* and *Itgb3*, which are known to regulate migration of trophectoderm cells. In particular, the expression of *Itgb3*, an integrin subunit gene, exhibits high inverse correlation with that of *Cdx2* in the TE. Reflecting the down-regulation of the genes for TE migration, TE-specific *Cdx2* overexpression causes suppression of the blastocyst outgrowth *in vitro* and abnormal progression of implantation *in vivo*. Thus, our results specify the time-course changes of global gene expression in the mTE during implantation and uncover the significance of *Cdx2* down-regulation for implantation progression.

## Introduction

Implantation, a process connecting an embryo to a mother, is essential for mammalian development. In humans, the natural conception rate per menstrual cycle is limited to about 30% and pregnancy losses are mostly attributed to implantation failure (Norwitz et al., 2001). In addition, the low efficiency of implantation success is a bottleneck for infertile patients who use assisted reproductive technology (ART) to become pregnant (Cha et al., 2012). Thus, improvement of the implantation efficiency is one of the most important goals for enhancement of successful childbirth.

Blastocyst is constituted of an inner cell mass (ICM) and trophectoderm (TE), which is the origin of embryonic and extraembryonic tissues, respectively. Moreover, the TE is subdivided into the polar TE (pTE) contacting the ICM and the mural TE (mTE) surrounding the blastocoel (Rossant and Tam, 2009). In mice, the pTE cells remain in the undifferentiated state and form the extra-embryonic ectoderm (ExE) and ectoplacental cone (EPC) during implantation, and, after implantation, differentiate into three kinds of trophoblasts: spongiotrophoblasts, labyrinthine trophoblasts and secondary trophoblast giant cells (TGCs), which constitute the placenta (Sutherland, 2003; Latos and Hemberger, 2016). On the other hand, the mTE differentiates into the primary TGCs during implantation and the primary TGCs form a network of anastomotic channels surrounding embryos known as the yolk sac (Sutherland, 2003). Owing to the establishment of the trophoblast stem cells (TSCs) in mice from the pTE and ExE, various regulatory mechanisms of placental development have been elucidated (Tanaka et al., 1998; Latos and Hemberger, 2016). In contrast, understanding of the differentiation mechanisms of the mTE has been delayed by the lack of an appropriate *in vitro* model.

In humans and rodents, the processes of implantation consist of three stages: apposition, attachment and invasion (Dey et al., 2004). After the mouse embryo develops to the blastocyst stage by embryonic day (E) 3.5, the blastocyst moves in close proximity to and contacts the uterine luminal epithelium (apposition) between E3.5 and E4.0, connects to the uterine tissue between E4.0 and E4.5 (attachment) and begins to invade the uterine tissue from E5.0 (Egashira and Hirota, 2013; Matsumoto, 2017). Since these processes are initiated from the mTE, the acquisition of adhesion and migration ability in the mTE is critical for the implantation progression (Sutherland, 2003). Some characteristic changes of the mTE which allow it to adhere to and invade the endometrium have already been identified, such as the activation of integrin signaling, the phagocytosis-like mechanism and the vascular-like gene expression patterns (El-Shershaby and Hinchliffe, 1975; Parr et al., 1987; Armant, 2005; Chaen et al., 2012; Li et al., 2015; Govindasamy et al., 2021). However, the overall picture and time-course of the characteristic changes of the mTE, and their regulatory mechanisms remain largely unclear.

*Cdx2* is a member of the caudal-related homeobox transcription factor gene family and well known to involve in placental development. CDX2 is initially detected in morula-stage embryos and become restricted to outer cells (TE progenitors) by the blastocyst stage (Posfai et al., 2017). In these stages, CDX2 contributes to the repression of the pluripotent gene expression program and the acquisition of TE cell fate (Niwa et al., 2005; Strumpf et al., 2005). On the other hand, in post-implantation stage, CDX2 expression is required to maintain the stemness of pTE-derived trophoblasts for placental formation and decreases along with its differentiation (Latos et al., 2015a; Suzuki et al., 2019; Saha and Ain, 2020). Thus, the functions of CDX2 are different in extraembryonic cells before and after implantation. Recently, we reported that CDX2 expression decreased in the mTE but not in the pTE during implantation (Suzuki et al., 2022). These results suggest that CDX2 has important functions for implantation such as the repression of TE differentiation. However, the exact functions of *Cdx2* during the peri-implantation stage remains unclear.

In the present study, RNA sequencing (RNA-seq) analysis was performed with the mTE of pre-implantation (E3.5), peri-implantation (E4.0 and 4.5) and non-implanting in vitro-cultured blastocysts to understand stage-specific changes in gene expression of the mTE during implantation. In addition, we performed TE-specific *Cdx2* overexpression by lentiviral vector-mediated gene transduction to explore the significance of *Cdx2* expression for mTE differentiation and implantation progression.

## Materials and Methods

### Animals and Embryo Collection

C57BL/6N, DBA/2J and ICR mice were purchased from CLEA Japan and maintained in accordance with the Guidelines for the Care and Use of Laboratory Animals, as specified by the Japanese Association for Laboratory Animal Science and by the Tokyo University of Agriculture (approval number: 2020018). BDF1 background blastocysts for RNA-seq analysis were generated by natural mating between adult female C57BL/6N mice and male DBA/2J mice and collected by flushing uteri in M2 medium at E3.5, 4.0, and 4.5. Some of the E3.5 blastocysts were cultured in KSOM for 24 h to prepare E3.5 + 24 h blastocysts. Blastocysts for immunofluorescence analysis were generated by natural mating between adult ICR mice and collected by flushing uteri in M2 medium. For LV vector transduction experiments, ICR and BDF1 (C57BL/6N × DBA/2J) mice were purchased from CLEA Japan or Japan SLC and maintained in accordance with the guidelines specified by the Animal Care and Use Committee of Gunma University (approval number: 21-025). Adult female mice were superovulated by intraperitoneal injection of 7.5 IU of pregnant mare serum gonadotropin (PMSG) (ASKA Pharmaceutical, Tokyo), and, 48 h later, 7.5 IU of human chorionic gonadotropin (hCG) (ASKA Pharmaceutical). MII oocytes were collected from the oviducts 15 h after hCG injection and used for *in vitro* fertilization (IVF) with sperm collected from adult male mice in HTF medium. The embryos acquired by IVF were cultured in EmbryoMax Advanced KSOM Embryo Medium (Advanced KSOM; Merck) for about 80 h to develop them to blastocysts. ICR and BDF2 background blastocysts were used for outgrowth and ET experiments, respectively. To produce pseudo-pregnant mice for ET, adult female ICR mice were mated with vasectomized male ICR mice.

### Preparation of Samples and RNA-Seq

For RNA-seq analysis of the mTE, after collecting the E3.5, 4.0, 4.5, and E3.5 + 24 h blastocysts, each blastocyst was cut into a polar part containing the ICM and the pTE, and a mural part containing only the mTE by a microblade (Bio-Cut Blade No. 715; Feather) attached to a micromanipulator. The zona pellucida of E3.5 and E3.5 + 24 h blastocysts was removed by acidic Tyrode’s solution (Sigma-Aldrich) before cutting. The mTE isolated from the blastocysts was immediately lysed in Buffer RLT Plus from an RNeasy Plus Micro Kit (QIAGEN) on ice. The mTE collected from 16 blastocysts was pooled as a sample to measure the quality of RNA using Bioanalyzer (Agilent) after total RNA extraction, and three samples were prepared for each stage. Total RNA of the mTE was extracted using an RNeasy Plus Micro Kit according to the manufacturer’s instructions. Complementary DNA (cDNA) libraries were generated from total RNA with high RNA integrity number (RIN) values (RIN >7) using a SMART-seq HT PLUS Kit (Clontech) according to the manufacturer’s instructions.

For RNA-seq analysis of the LV vector-transduced blastocysts, LV-*Egfp*- and LV-*Cdx2*-transduced blastocysts that had been transferred into pseudo-pregnant mice were collected by flushing uteri in M2 medium at 4.5 dpc. Six LV-*Egfp*-transduced blastocysts and eight LV-*Cdx2*-transduced blastocysts were individually lysed in Buffer RLT Plus. Total RNA of the blastocysts was extracted using an RNeasy Plus Micro Kit and cDNA libraries were generated from half of the extracted total RNA using a SMART-seq HT PLUS Kit according to the manufacturer’s instructions. The indexed libraries were sequenced using an Illumina NextSeq 500 under 75-bp single-end conditions.

### RNA-Seq Data Processing and Bioinformatic Analysis

Alignment of sequence reads to the *Mus musculus* genome, Genome Reference Consortium Mouse Build 39 (mm39), and gene expression quantification were performed using an integrated sequence analysis software package, CLC Genomics Workbench (Filgen, Nagoya, Japan). For correlation analysis, PCA, clustering analysis and extraction of differentially expressed genes (DEGs), the genes with maximum reads per kilobase of transcript per million reads (RPKM) values ≥10 in at least one sample were used. Spearman’s correlation analysis was performed using the “cor” function of the R package. PCA was performed using the “prcomp” function of the R package with log2-transforming RPKM values (log_2_ (RPKM+1)). Hierarchical clustering was performed based on Ward’s method using the “hclust” function of the R package. The DEGs between each stage or condition were defined as genes exhibiting fold-change ≥ 2 and FDR *p*-value ≤ 0.01 on a CLC Genomics Workbench. The DEGs between each stage of the mTE were classified into k-means clusters (K = 9) using the “kmeans” function of the R package with Z-score values. Heatmaps were generated using the “heatmap.2” function of the R package with correlation coefficient, Z-score or log2-transforming RPKM values (log_2_ (RPKM+1)). Venn diagrams were generated using the “venn.diagram” function of the R package, and common and specific genes between the gene lists were extracted using the “intersect” and “setdiff” functions, respectively. The gene ontology (GO) and Kyoto Encyclopedia of Genes and Genomes (KEGG) pathway analyses were performed using the DAVID web tool.

### Lentiviral Production and Titration

pLV-EF1A-EGFP, a lentiviral vector plasmid enabling constitutive expression of *Egfp*, was designed by and purchased from VectorBuilder. The mouse *Cdx2* CDS sequence was amplified by RT-PCR using BDF1 TSCs previously established by our group (Ogawa et al., 2015). pLV-EF1A-CDX2, a lentiviral vector plasmid enabling constitutive expression of *Cdx2*, was produced by replacing the EGFP sequence of pLV-EF1A-EGFP with a mouse *Cdx2* CDS sequence using In-Fusion Snap Assembly Master Mix (Clontech). The lentiviral vector plasmids were transfected to 293T cells with lentiviral packaging plasmids (BiOSETTIA) by the calcium phosphate method in DMEM supplemented with 10% fetal bovine serum (FBS), 50 U/ml penicillin (Gibco), and 50 μg/ml streptomycin (Gibco) (DMEM + FBS). On the following morning, the medium was changed to DMEM + FBS supplemented with 10 μM forskolin. Lentiviral vectors were harvested 48 h after the medium change and concentrated by ultracentrifugation (50,000×g, 2 h × 2 times). After resuspension with PBS, the lentiviral vectors were washed and further concentrated using an Amicon Ultra-4 filter unit (Merck). The concentration of the lentiviral vectors was determined by a Lenti-X qRT-PCR Titration Kit (Takara Bio) according to the manufacturer’s instructions.

### Outgrowth Assay of Lentiviral Vector-Transduced Blastocysts

The zona pellucida of the blastocysts was removed by acidic Tyrode’s solution (Sigma-Aldrich). Zona pellucida-free blastocysts were incubated in groups of three or four in 5 μL of Advanced KSOM containing lentiviral vectors (3.125×10^8^, 6.25×10^8^ or 12.5×10^8^ copies/ml) for 5 h. The lentiviral vector-transduced blastocysts were cultured in 500 μL DMEM + FBS on gelatin-coated 4-well dishes. When the blastocysts were cultured for 48 and 72 h, the progression of outgrowth was evaluated in three stages: pre-outgrowth (stage 1), partial outgrowth (stage 2), and full outgrowth (stage 3). Stages 2 and 3 were distinguished according to whether blastocoel cavity was collapsed (stage 3) or not (stage 2).

### RT-qPCR

Total RNA was extracted from the outgrowth samples cultured for 72 h using an RNeasy Plus Micro Kit according to the manufacturer’s instructions. cDNA was synthesized from the whole quantity of the total RNA by using ReverTra Ace qPCR RT Master Mix (TOYOBO) according to the manufacturer’s instructions. qPCR was performed using Power SYBR Green PCR Master Mix (Applied Biosystems) and the target gene-specific primers on a QuantStudio three Real-time PCR System (Applied Biosystems). The relative expression level of each target gene was calculated by normalizing to *Gapdh*. The primer sequences are listed in **Supplementary Table1**.

### Embryo Transfer of Lentiviral Vector-Transduced Blastocysts

Six or seven of the lentiviral vector-transduced blastocysts were transferred into each horn of the uterus of pseudo-pregnant mice at 2.5 dpc. To visualize the implantation sites of the transferred blastocysts, 200 μL of 1% Chicago sky blue dye (Santa Cruz) in PBS was intravenously injected into the recipient mice at 4.5 or 5.5 dpc. Uteri were dissected from the mice 5 min after injection, and the blue bands were counted as the implantation sites. For RNA-seq analyses, the transferred blastocysts were collected by flushing uteri in M2 medium at 4.5 dpc.

### Whole-Mount Immunofluorescence Staining

Embryos were fixed with 4% paraformaldehyde (PFA) in PBS for 30 min at RT and permeabilized with 0.2% Triton X-100 in 0.1% BSA/0.1% PVA/PBS (wash solution) for 15 min at RT. After the non-specific antigens were blocked with 2% FBS or donkey serum/0.1% Tween20/wash solution for 2 h at RT, embryos were incubated in blocking solution with primary antibodies overnight at 4°C. Primary antibodies were labeled with Alexa Fluor-conjugated secondary antibodies diluted in blocking solution for 1 h at RT. The embryos were transferred onto a glass bottom dish (Matsunami or MatTek) containing drops of vectashield with DAPI (Vector Laboratories) diluted with wash solution (1:10), and then the immunofluorescence images were obtained using a confocal laser scanning microscopy (LSM710; Carl Zeiss, Oberkochen, Germany).

The images were analyzed with LSM software ZEN 2011 and Fiji software (RRID:SCR_002285, https://fiji.sc/#) (Schindelin et al., 2012). Single-plane or Z-stack images (2 μm intervals) were obtained with Plan Apochromat 20x/0.8 or Plan Apochromat 40x/1.4 oil objectives. To analyze Ki67 fluorescence intensity of polar and mural TE, single-plane images of the mid-section of the blastocysts that included the ICM and blastocoel were obtained. Manual nuclear segmentation along DAPI fluorescence and quantification of CDX2 fluorescence intensity were performed using ROI Manager in the Fiji software. To compare the fluorescence intensity between pTE and mTE, blastocysts were divided into three equal segments (polar, intermediate, mural) along the embryonic-abembryonic axis, and the fluorescence intensity in each of the cells included in polar and mural segments was measured. The intermediate segments were excluded from the analysis. The fluorescence intensity was quantified by averaging pixel intensities in each nucleus.

The primary and secondary antibodies used in the present study are listed in **Supplementary Table2**.

### Hematoxylin and Eosin Staining and Immunohistochemistry

Uterine tissues were fixed in 10% formalin (FUJIFILM Wako) overnight, dehydrated in a graded series of ethanol solutions (70%, 80%, 90% and 100% ethanol) and xylene, embedded in paraffin, and sectioned at 5 μm intervals. Paraffin sections were hydrated in xylene and a graded series of ethanol solutions (100%, 90%, 80% and 70% ethanol) for subsequent hematoxylin and eosin (H&E) staining and immunohistochemistry (IHC). H&E staining was performed with the hydrated sections according to standard protocols. For IHC, antigen retrieval was performed by boiling in 10 mM sodium citrate buffer (pH6.0) for 15 min, and endogenous peroxidase was blocked with 0.3% H_2_O_2_ diluted in methanol for 30 min. After blocking with 3% BSA/PBS, the sections were incubated in 3% BSA/PBS with primary antibodies overnight at 4°C, 3% BSA/PBS with biotinylated secondary antibodies for 30 min at RT, and avidin-biotinylated horseradish peroxidase complex (VECTASTAIN Elite ABC Kit; Vector Laboratories) for 30 min at RT. Positive signals were visualized with Histofine simple stain DAB solution (Nichirei) and counterstaining was performed with hematoxylin. The primary antibodies used in the present study are listed in **Supplementary Table2**.

### Statistical Analysis

RT-qPCR data are presented as mean ± S.D. Student’s *t*-test was used for comparison between two groups. Dunnett test was used for multiple comparisons among experimental groups. Fisher’s exact test was used for analysis of qualitative data. Association of gene expression levels was analyzed by Spearman’s correlation coefficient with log2-transforming RPKM values (log_2_ (RPKM+1)). Each experiment included at least three independent samples. *p* values less than 0.05 were considered statistically significant.

## Results

### Transcriptomic Dynamics of the mTE During Implantation

**FIGURE 1:**
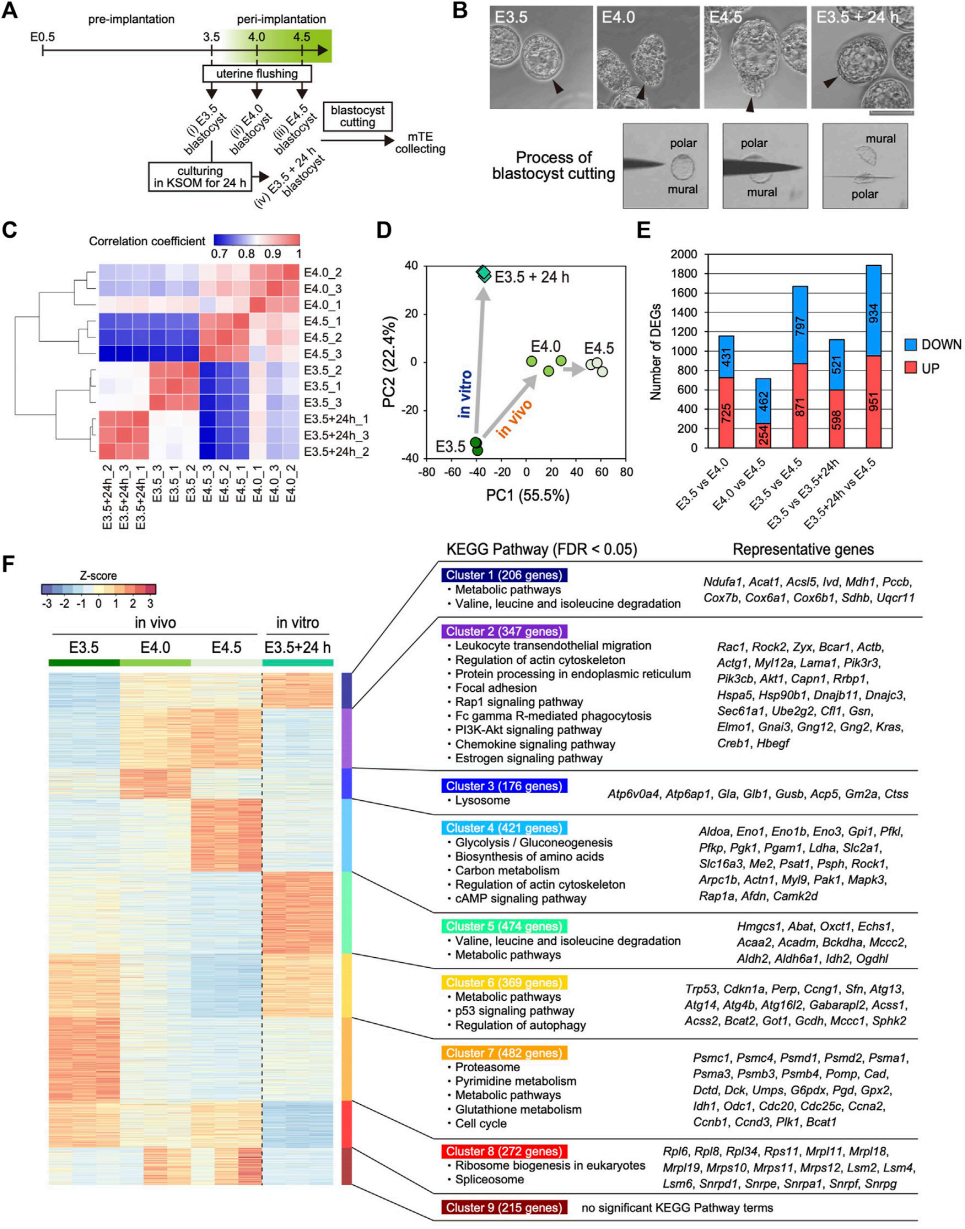
Transcriptomic profiling of the mTE at E3.5, E4.0 and E4.5 **(A)** Schemes for the production of *Egfp*-OE and *Cdx2*-OE blastocysts, and the assessment of implantation ability *in vitro* and *in vivo* **(B)** Images of the prepared blastocysts (top) and successive images of the mTE isolation by a microblade (bottom). Arrowheads indicate the tips of the mTE. Scale bar: 100 µm **(C)** Heatmap showing Spearman’s correlation of RNA-seq replicates **(D)** Principal component analysis (PCA) of the E3.5, E4.0, E4.5 and E3.5 + 24 h mTE **(E)** The number of DEGs between each stage of the mTE **(F)** Heatmap of k-means non-hierarchical clustering and the KEGG Pathway with significant enrichment of genes included in each cluster.

To elucidate the time-course changes in the transcriptome of the mTE during implantation, we performed RNA-seq analysis of multiple stages of the mTE. Pre- (E3.5) and peri-implantation (E4.0 and 4.5) blastocysts were collected by flushing uteri (Figure 1A). We previously reported that the blastocyst culturing in KSOM maintains the expansion of the blastocoel and does not proceed with outgrowth (Suzuki et al., 2022). In the present experiments, therefore, some of the E3.5 blastocysts were cultured in KSOM for 24 h to prepare *in vitro* E4.5 blastocysts as non-implanting blastocysts that we termed E3.5 + 24 h blastocysts (Figure 1A). Morphologically, while E3.5 blastocysts have the TE with an epithelial-like flat surface, the mTE of E4.0 and E4.5 blastocysts appears lumpy. On the other hand, E3.5 + 24 h blastocysts exhibit the expanded blastocoel and the TE with an epithelial-like surface, as previously described (Figure 1B) (Suzuki et al., 2022). These four kinds of blastocysts (E3.5, E4.0, E4.5 and E3.5 + 24 h) were cut into polar and mural parts by a microblade attached to a micromanipulator, and only the mTE was collected as RNA-seq samples (Figure 1A,B). Expression analysis of the TE marker CDX2 and ICM marker SOX2 by immunofluorescence and RT-PCR confirmed that the mTE could be correctly isolated without contamination by the cells on the pTE side (Supplementary Figure S1A,B). We pooled the mTE collected from 16 blastocysts as one sample, prepared three samples for each stage and subjected them to RNA-seq analysis. We first analyzed the expression of epiblast and primitive endoderm (PrE) marker genes to ensure ICM cells were not present in the samples. The expression levels of the representative epiblast marker genes *Sox2*, *Esrrb*, *Fgf4* and *Nr0b1*, and the representative PrE marker genes *Gata4*, *Sox7*, *Sox17* and *Pdgfra* were considerably low in all samples (RPKM <10) (Supplementary Figure S2). Especially, the transcripts of *Sox2*, an early ICM marker gene, whose expression is completely restricted to the ICM at early blastocyst stage (Guo et al., 2010; Wicklow et al., 2014), were hardly detected in all samples (RPKM <0.1) (Supplementary Figure S2). These results ensured the mTE was correctly isolated and collected as RNA-seq samples without containing the ICM cells. On the other hand, interestingly, the expression of some epiblast marker genes, such as *Pou5f1*, *Nanog* and *Tbx3*, and the PrE marker gene *Dab2* was strongly detected at E3.5, and the expression levels of these genes decreased over time from E3.5–4.5 *in vivo* (Supplementary Figure S2). Thus, it was suggested that the transcriptional character of the ICM is partially retained in the TE at the pre-implantation stage and disappears during implantation.

Next, we analyzed the changes in the global gene expression of the mTE during implantation. Spearman’s correlation coefficient analysis showed that the global gene expression changed more greatly in *in vivo* than *in vitro*, especially from E3.5–4.0 (Figure 1C). The number of differentially expressed genes (DEGs: fold change ≥2, FDR *p*-value ≤ 0.01) also indicated that the gene expression changed more greatly between E3.5 and E4.0 than between E4.0 and E4.5 (Figure 1E). These results suggested that the interaction between the blastocyst and uterine tissue has a major impact on the gene expression of the mTE. Remarkably, the DEGs over E3.5–4.0 and E4.0–4.5 did not include many common genes, and those specific to E3.5–4.0 and E4.0–4.5 were preferentially enriched for the different gene ontology (GO) terms (Supplementary Figure S3A). Principal component analysis (PCA) indicated that the pattern of change in global gene expression in the mTE *in vivo* differed from that *in vitro* (Figure 1D), which was further confirmed by the results that in vivo- and in vitro-specific DEGs were preferentially enriched for the different GO terms (Supplementary Figure S3B). To gain insights into the changes in the characteristics of the mTE during implantation, we classified all the DEGs during each stage into nine groups according to their expression patterns by k-means non-hierarchical clustering and performed Kyoto Encyclopedia of Genes and Genomes (KEGG) Pathway analysis with the genes included in each cluster (Figure 1F). As a result, surprisingly, it was shown that the mTE dynamically changed its gene expression for a short period of time from E3.5 to E4.5. Furthermore, it was revealed that the changes in gene expression *in vivo* were highly different from those *in vitro*. Specifically, the genes for cell adhesion and migration (“Regulation of actin cytoskeleton”, “Focal adhesion” and “Rap1 signaling pathway”), and phagocytosis (“Fc gamma R-mediated phagocytosis” and “Phagosome”) are up-regulated from E4.0 *in vivo* but not at E3.5 + 24 h, whereas those for “Metabolic pathways”, “p53 signaling pathways” and “Regulation of autophagy” are down-regulated from E4.0 *in vivo*. Moreover, the genes for “Lysosome” are transiently up-regulated at E4.0 and those for “Glycolysis”, “Biosynthesis of amino acids”, “Carbon metabolism” and “cAMP signaling pathway” are up-regulated at E4.5 *in vivo*. On the other hand, the genes for “Valine, leucine and isoleucine degradation” and “Metabolic pathways” are up-regulated at E3.5 + 24 h, whereas those for “Ribosome” and “Spliceosome” are down-regulated. Furthermore, the genes for “Proteasome”, “Pyrimidine metabolism”, “Metabolic pathways”, “Glutathione metabolism” and “Cell cycle” are down-regulated from E4.0 *in vivo* as well as at E3.5 + 24 h. Therefore, it was suggested that the characteristics of the mTE are dramatically and stage-specifically varied in order to allow implantation to proceed in an orderly manner.

### Differentiation of the mTE During Implantation

**FIGURE 2:**
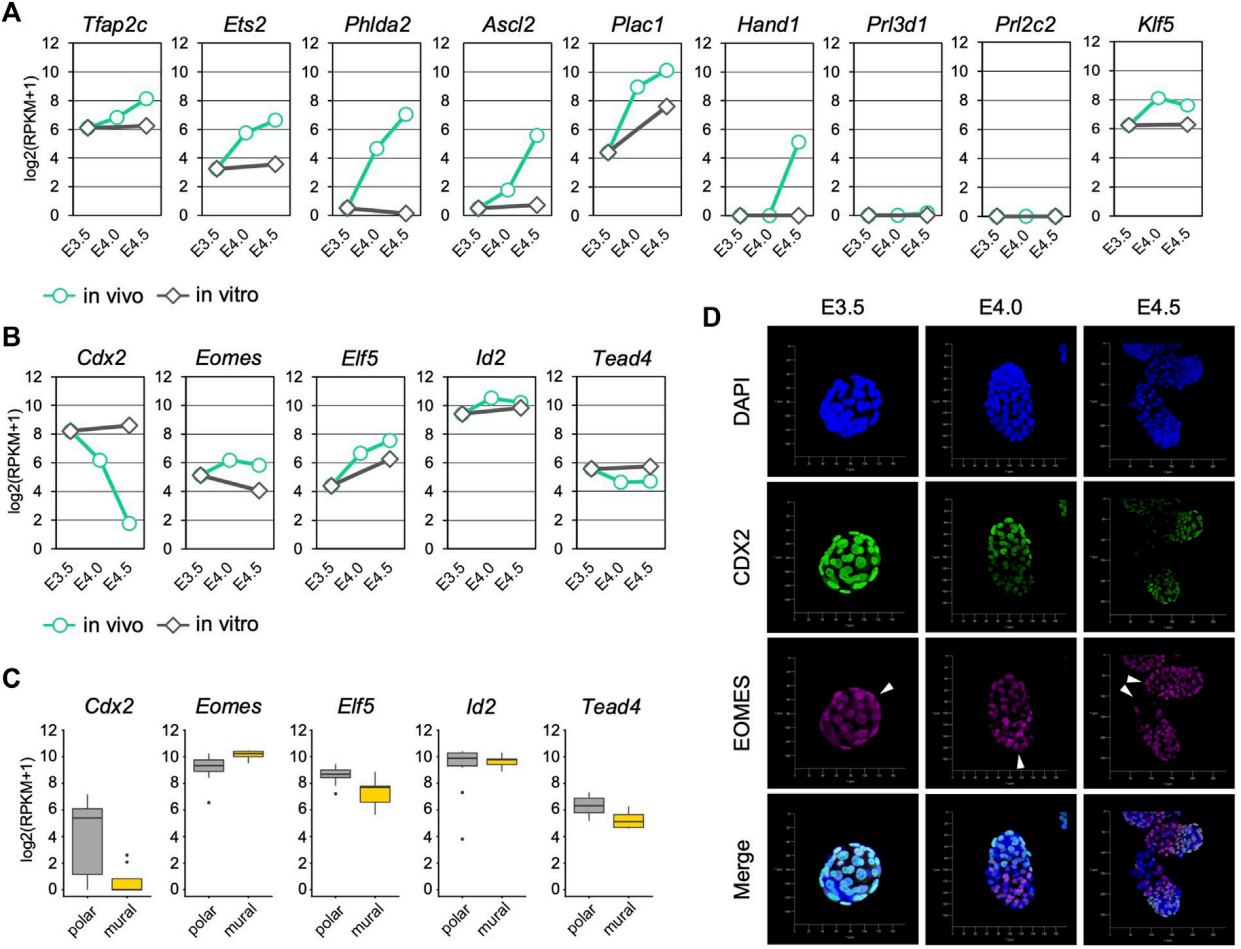
Trophoblast marker gene expression in the mTE during implantation **(A)** mRNA levels of differentiation marker genes detected by RNA-seq analysis in mTE **(B)** mRNA levels of undifferentiation marker genes detected by RNA-seq analysis in mTE **(C)** mRNA levels of undifferentiation marker genes in each cell of the pTE and mTE. These results were obtained by re-analyzing the published single-cell RNA-seq datasets of E4.5 blastocysts **(D)** Immunostaining of CDX2 and EOMES in blastocysts at E3.5, E4.0 and E4.5. Arrowheads indicate the tips of the mTE. Scale bar: 50 µm.

We next analyzed the expression of trophoblast differentiation marker genes to evaluate the progression of mTE differentiation during implantation. The differentiation marker genes *Tfap2c*, *Ets2*, *Phlda2* and *Ascl2* were up-regulated over time from E3.5 to E4.5 *in vivo* but not *in vitro* (Figure 2A). *Klf5* that regulates TE development cell-autonomously was also up-regulated *in vivo* (Lin et al., 2010). *Plac1* was up-regulated both *in vivo* and *in vitro*, but the expression level at E3.5 + 24 h was about 4-fold lower than that at E4.5 (Figure 2A). Interestingly, the expression of *Hand1*, which promotes trophoblast differentiation into TGCs (Riley et al., 1998; Scott et al., 2000), was detected at E4.5, although it was not present at E3.5, E4.0 or E3.5 + 24 h (Figure 2A). In contrast, the expression of TGC marker genes *Prl3d1* and *Prl2c2* was hardly detected at any stage (RPKM <1) (Figure 2A). These results indicated that differentiation of the mTE into the primary TGCs was initiated at least from E4.0 and completed later than E4.5 *in vivo*, but it did not progress *in vitro*. It has been reported that mouse trophoblasts stop mitosis and restart DNA synthesis without the mitotic phase (endoreduplication) during differentiation to become polyploid TGCs (Ullah et al., 2009; Hu and Cross, 2010). We therefore analyzed the expression of cell cycle-related genes in the mTE. Many genes accelerating the cell cycle, including the genes encoding cyclins and cyclin-dependent kinases, were down-regulated from E4.0, suggesting that the cell cycle began to be arrested in the mTE from E4.0 (Supplementary Figure S4A). In fact, immunofluorescence analysis showed that the Ki67 protein, which is expressed throughout the entire cell cycle except in the G_0_ phase, decreased from E4.0 and disappeared at E4.5 in the mTE but not the pTE (Supplementary Figure S5). These results indicated that the mTE arrested the cell cycle in association with the progression of differentiation. Interestingly, genes regulating DNA replication, such as the genes encoding DNA polymerases and minichromosome maintenance protein (MCM) complex components, were down-regulated at E4.0 and then up-regulated again at E4.5 to the same levels as at E3.5 (Supplementary Figure S4B). Moreover, *Ccne*, a cyclin that initiates DNA replication, were up-regulated at E4.5 (Supplementary Figure S4A). Thus, it is possible that the mTE began to arrest mitosis by E4.5, and then re-activated the gene expression promoting DNA replication to induce endoreduplication from E4.5. Considering also that *Hand1* expression began to be detected at E4.5, it was suggested that the mTE starts to acquire the TGC transcriptional program from E4.5.

We also analyzed the expression of trophoblast undifferentiation marker genes. *Cdx2* was down-regulated over time *in vivo*, but its expression was maintained *in vitro* (Figure 2B). Although *Tead4* was also down-regulated *in vivo*, the extent of its down-regulation was far smaller than that of *Cdx2* (Figure 2B). In contrast, *Eomes*, *Elf5* and *Id2* did not exhibit down-regulation like that of *Cdx2* either *in vivo* or *in vitro* (Figure 2B). We also analyzed the transcriptomic dataset of a single TE dissected from E4.5 blastocysts (Nakamura et al., 2015) and found that the expression level of *Cdx2* in the mTE was much lower than that in the pTE, whereas other undifferentiated marker genes did not show such large difference (Figure 2C). Immunofluorescence analysis showed that CDX2 expression was gradually decreased from E3.5 to E4.5 and completely repressed at E4.5 in the mTE as previously described (Suzuki et al., 2022). However, EOMES was detected in the mTE at E4.0 at a similar level as in the pTE. Even at E4.5, cells strongly expressing EOMES were found in the mTE (Figure 2D). Thus, it was indicated that *Cdx2* was down-regulated prior to other undifferentiation marker genes during mTE differentiation. Taken together, we showed that differentiation of the mTE was initiated at least from E4.0 *in vivo* and the expression patterns of undifferentiation marker genes in the mTE might be different from those in the pTE-derived trophoblasts.

### *Cdx2* Overexpression Hinders the Development of Blastocyst Outgrowth

**FIGURE 3:**
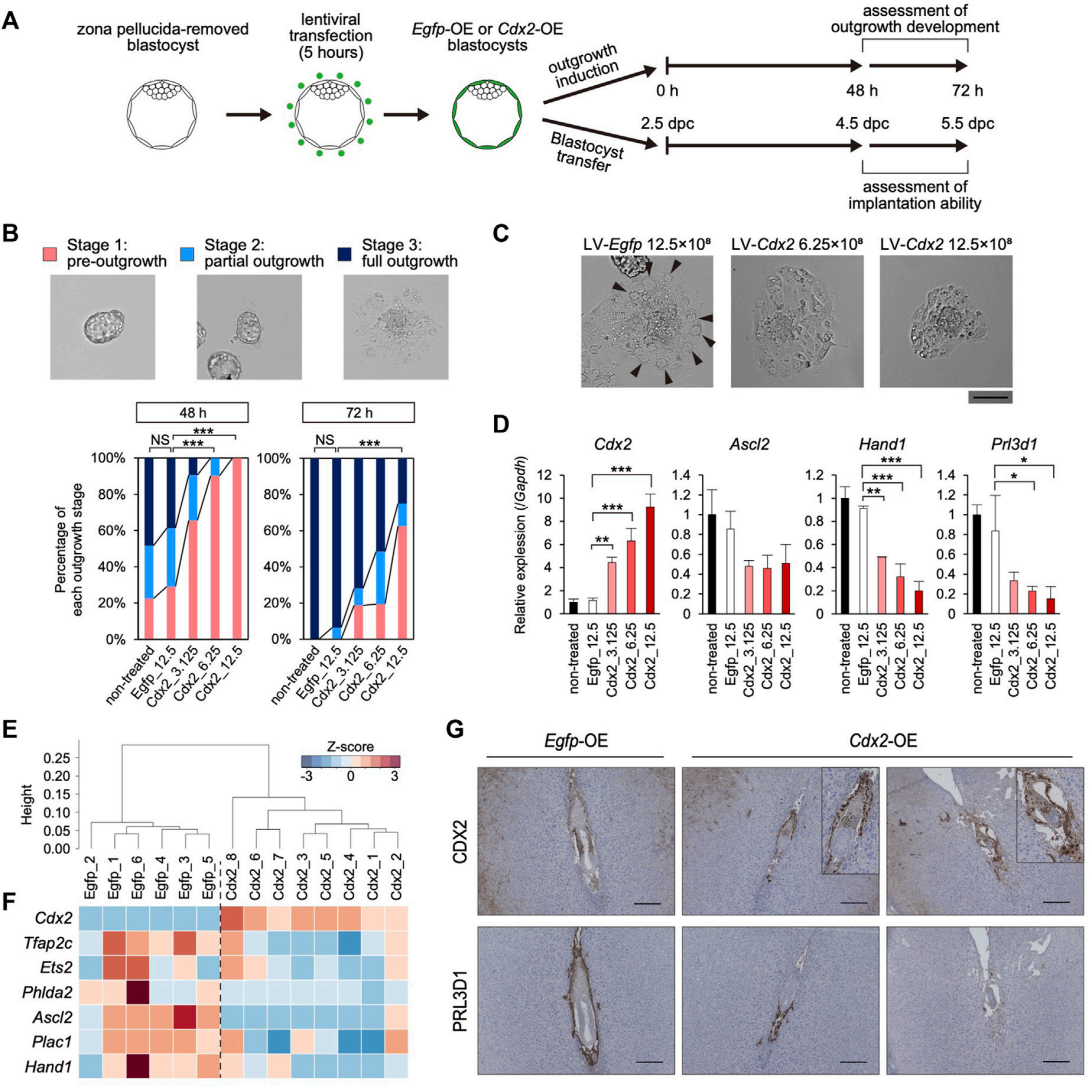
The effect of TE-specific *Cdx2* overexpression on differentiation of the mTE **(A)** A scheme for the production of *Egfp*-OE and *Cdx2*-OE blastocysts, and the subsequent experiments: outgrowth induction and *in vivo* development after transfer into uteri **(B)** Representative images and percentage of embryos in each outgrowth stage. Fisher’s exact test; ***p < 0.001. NS: not significant **(C)** Higher magnification images of blastocyst outgrowth. Arrowheads indicate the cells showing TGC-like morphology. Scale bar: 100 µm **(D)** RT-qPCR analysis of *Cdx2* and differentiation marker genes (*Ascl2*, *Hand1* and *Prl3d1*) in blastocyst outgrowth. The values are normalized to *Gapdh* and indicated as the mean ± S.D. (n = 3). Dunnett test; *p < 0.05, **p < 0.01, ***p < 0.001 **(E)** Hierarchical clustering analysis based on global gene expression **(F)** Heatmap showing the expression levels of *Cdx2* and differentiation marker genes. In particular, *Cdx2*, *Ascl2* and *Phlda2* were identified as DEGs (fold-change ≥ 2, FDR *p*-value ≤ 0.01) **(G)** *Egfp*-OE and *Cdx2*-OE E5.5 conceptuses labelled for CDX2 or PRL3D1. Boxed regions are shown at higher magnification of conceptuses.

Given that *Cdx2* expression decreased along with the up-regulation of the differentiation marker genes in the mTE, we hypothesized that *Cdx2* expression regulates mTE differentiation. Therefore, we overexpressed *Cdx2* in a TE-specific manner by means of lentiviral (LV) vector-mediated gene transduction (Okada et al., 2007). Pre-implantation blastocysts from which the zona pellucida had been removed in acidic Tyrode’s solution were transduced with the LV vectors harboring *Cdx2* expression cassette (LV-*Cdx2*). We also prepared blastocysts transduced with LV vectors harboring *Egfp* expression cassette (LV-*Egfp*) as a control. These blastocysts were cultured in DMEM supplemented with 10% FBS (DMEM + FBS) on gelatin-coated dishes to induce blastocyst outgrowth, and then their outgrowth development was compared to that of blastocysts without LV vector transduction (non-treated control) (Figure 3A). Immunofluorescence analysis showed that CDX2 expression was increased in the TE of LV-*Cdx2*-tranduced blastocysts in a manner dependent on LV-*Cdx2* concentration, but was not increased in LV-*Egfp*-transduced blastocysts compared to the non-treated control blastocysts after 24 h (Supplementary Figure S6A). Outgrowth development was evaluated in three stages: pre-outgrowth (stage 1); partial outgrowth (stage 2); and full outgrowth (stage 3) (Figure 3B). We distinguished between stage 2 and 3 according to whether the blastocoel cavity was completely collapsed (stage 3) or not (stage 2). After outgrowth induction for 48 h, while more than 70% of the non-treated control and LV-*Egfp*-transduced blastocysts initiated outgrowth (stage 2 or 3), only less than 40% of the LV-*Cdx2*-transduced blastocysts initiated outgrowth. After outgrowth induction for 72 h, while all of the non-treated control and LV-*Egfp*-transduced blastocysts initiated outgrowth, LV-*Cdx2* transduction reduced the rate of stage 2 and 3 outgrowth to 40–80%. In particular, transduction with LV-*Cdx2* at 12.5×10^8^ copies/ml significantly reduced the rate of stage 2 and 3 outgrowth even 72 h after outgrowth induction (Figure 3B, Supplementary Figure S7A). These results indicated that *Cdx2* overexpression delayed outgrowth development by repressing migration of the TE cells. Importantly, part of LV-*Cdx2*-transduced blastocysts reached stage 3, but a smaller number of cells with TGC-like morphology appeared in the stage 3 outgrowth compared to that derived from the non-treated control and LV-*Egfp*-transduced blastocysts (Figure 3C). We then analyzed the expression of differentiation marker genes in outgrowth-induced samples to reveal whether LV-*Cdx2* transduction interfered with differentiation of the mTE during outgrowth. The results showed that *Cdx2* expression was significantly increased in the LV-*Cdx2*-transduced samples in an LV-*Cdx2* concentration-dependent manner (Figure 3D). In contrast, expression of the early differentiation marker gene *Ascl2* decreased by about 2-fold and that of the late differentiation marker genes *Hand1* and *Prl3d1* decreased by more than 2-fold in *Cdx2*-LV-transduced samples compared to LV-*Egfp*-transduced samples (Figure 3D). These results indicated that *Cdx2* overexpression repressed TE differentiation during outgrowth. Thus, it was suggested that *Cdx2* overexpression hindered the development of blastocyst outgrowth by repressing TE differentiation and migration.

### *Cdx2* Down-Regulation Is Required for mTE Differentiation *in Vivo*

We also investigated the effect of TE-specific *Cdx2* overexpression on differentiation of the mTE during implantation. LV-*Egfp*- and LV-*Cdx2* (12.5×10^8^ copies/ml) -transduced blastocysts were transferred into the uteri of pseudo-pregnant mice at 2.5 days post-coitum (dpc), and recovered by flushing the uteri at 40 h after blastocyst transfer (4.5 dpc). Immunofluorescence analysis showed that CDX2 was strongly expressed in the mTE as well as the pTE of LV-*Cdx2*-transduced blastocysts (Supplementary Figure S6B). We performed RNA-seq analysis of the *Egfp*-overexpressed (OE) and *Cdx2*-OE blastocysts recovered from uteri to elucidate whether mTE differentiation was hindered. Six and eight blastocysts in each group (*Egfp*-OE and *Cdx2*-OE blastocysts) were collected and subjected to the analysis individually. In hierarchical clustering analysis, *Egfp*-OE and *Cdx2*-OE blastocysts were classified into different clusters (Figure 3E). *Cdx2* expression was increased in *Cdx2*-OE blastocysts compared to *Egfp*-OE blastocysts (Figure 3F). In contrast, expression of the differentiation marker genes *Tfap2c*, *Ets2*, *Phlda2*, *Ascl2*, *Plac1* and *Hand1* tended to decrease in *Cdx2*-OE blastocysts (Figure 3F). The extent of *Ascl2* and *Phlda2* downregulation were especially significant (fold-change ≥ 2, FDR *p*-value ≤ 0.01). These results indicated that *Cdx2* overexpression hindered the initiation of mTE differentiation *in vivo*.

Next, we explored whether mTE differentiation into the primary TGCs is hindered by *Cdx2* overexpression at 5.5 dpc. Intravenous injection of Chicago sky blue dye revealed that implantation rates (implantation sites/transferred blastocysts) were approximately equal between *Egfp*-OE and *Cdx2*-OE blastocysts (68.7% vs 62.9%), indicating that *Cdx2* overexpression did not affect the initiation of implantation (Supplementary Figure S8A). Histological analysis showed that hemorrhage with excessive vascular-like structures were observed in 83.3% (5/6) implantation sites of *Cdx2*-OE embryos while almost all implantation sites of *Egfp*-OE embryos did not exhibit hemorrhage (1/6 implantation sites) (Supplementary Figure S8B). It has been known that embryos are located in the anti-mesometrial region of the decidua at 5.5 dpc under normal conditions. And indeed, in our experiments 83.3% (5/6) *Egfp*-OE embryos were located in the anti-mesometrial region. However, only 16.7% (1/6) *Cdx2*-OE embryos were located in the anti-mesometrial region and the others were located in central or mesometrial region (Supplementary Figure S8B). These results suggested that *Cdx2* overexpression might interfere with the normal progression of implantation. Immunohistochemistry analysis revealed that CDX2 and PRL3D1 were normally localized to the extraembryonic ectoderm (ExE) and the primary TGCs, respectively, in *Egfp*-OE embryos (Figure 3G). On the other hand, in *Cdx2*-OE embryos, CDX2 was detected not only in the ExE but also in cells surrounding the embryos (Figure 3G). Moreover, PRL3D1 was restricted to a small portion of the cells surrounding the embryos (Figure 3G). Comparison of CDX2 and PRL3D1 localization in serial sections showed that their expression patterns were mutually exclusive. These results indicated that *Cdx2* overexpression hindered mTE differentiation into the primary TGCs, although the *Cdx2*-OE cells appeared to be located in the boundary between the maternal tissues and the embryos as with the primary TGCs. Thus, we corroborated that *Cdx2* down-regulation is required for mTE differentiation and normal progression of implantation.

### *Cdx2* Down-Regulation Is Required for the mTE to Acquire the Potential for Cell Adhesion and Migration

**FIGURE 4:**
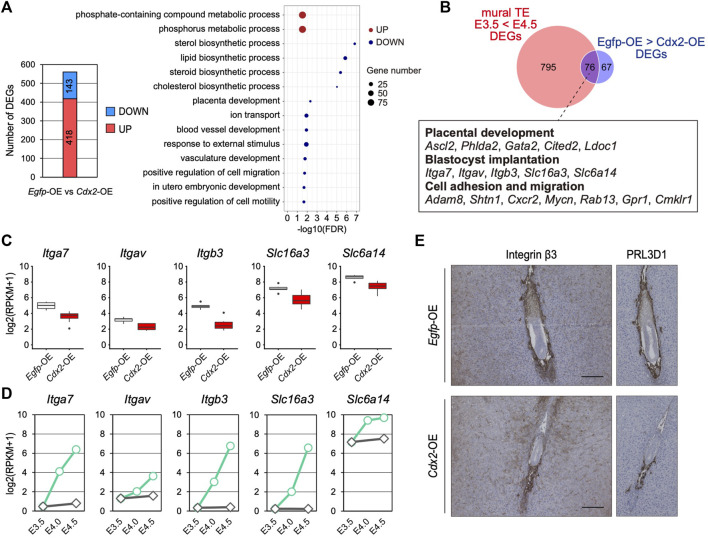
The effect of *Cdx2* on the mTE gene expression **(A)** The number of DEGs between *Egfp*-OE and *Cdx2*-OE blastocysts, and GO terms with preferential gene enrichment. In bubble plot, the size of each dot represents the number of genes enriched in the GO terms, while difference is shown by color: red, GO terms containing the up-regulated DEGs; blue, GO terms containing the down-regulated DEGs **(B)** Venn diagram showing the overlap of upregulated DEGs in the mTE at E4.5 compared to E3.5 and downregulated DEGs in LV-*Cdx2*-transduced blastocysts compared to LV-*Egfp*-transduced blastocysts **(C)** mRNA levels of *Itga7*, *Itgav*, *Itgb3*, *Slc16a3* and *Slc6a14* in *Egfp*-OE and *Cdx2*-OE blastocysts **(D)** mRNA levels of *Itga7*, *Itgav*, *Itgb3*, *Slc16a3* and *Slc6a14* in each stage of the mTE **(E)** E5.5 conceptuses labelled for ITGB3 and PRL3D1. The images of PRL3D1 staining were the same as in Figure 3G.

To further investigate the significance of *Cdx2* down-regulation in the mTE, we identified DEGs between *Egfp*-OE and *Cdx2*-OE blastocysts using transcriptomic profiles (Figure 4A). GO analysis showed that 143 DEGs down-regulated in *Cdx2*-OE blastocysts were enriched with genes for various biological processes such as “lipid biosynthetic process”, “placenta development”, “ion transport”, “blood vessel development”, “response to external stimulus” and “positive regulation of cell migration”, while 418 DEGs up-regulated in *Cdx2*-OE blastocysts were enriched with genes for “phosphate-containing compound metabolic process” (Figure 4A). Next, to extract candidate genes whose up-regulation may be induced by *Cdx2* down-regulation in the mTE, we compared the DEGs up-regulated at E4.5 vs E3.5 in the mTE (871 genes) and the DEGs down-regulated in *Cdx2*-OE blastocysts vs *Egfp*-OE blastocysts (143 genes). The intersection between these two sets of DEGs consisted of 76 genes. These common DEGs included the genes related to cell adhesion and migration, in addition to placental development, although they were not significantly enriched for any GO terms (Figure 4B–D). Among the cell adhesion and migration-related genes, *Itgav*, *Itga7*, *Itgb3*, *Slc6a14* and *Slc16a3* are known to regulate implantation in mice (Illera et al., 2000; Klaffky et al., 2001; Rout et al., 2004; Van Winkle et al., 2006; González et al., 2012; Gardner, 2015), suggesting that *Cdx2* overexpression interfered with the acquisition of adhesion and migration ability in the mTE. We then analyzed the transcriptomic dataset of a single TE dissected from E4.5 blastocysts (Nakamura et al., 2015) and found that there were inverse correlations (*p* < 0.05, r > 0.4) between the expression of *Cdx2* and the expressions of *Itgav*, *Itgb3*, *Itga7* and *Slc6a14* (Supplementary Figure S9). Among these genes, the expression of *Itgb3* had the highest inverse correlation (r = -0.840) with *Cdx2* expression (Supplementary Figure S9). *Itgb3* encodes integrin β3, which forms a heterodimeric complex with integrin αV or αIIb. Integrin αVβ3 and αIIbβ3 are the receptors for extracellular matrix proteins containing the RGD peptide motif, such as fibronectin, osteopontin and vitronectin (Hynes, 2002). Functional inhibition of integrin β3 using neutralizing antibodies represses TE migration during blastocyst outgrowth *in vitro* and hinders implantation *in vivo* (Illera et al., 2000; Rout et al., 2004). Thus, integrin β3 is critical for migration of the mTE. We then compared the expression of integrin β3 in *Egfp*-OE and *Cdx2*-OE embryos at 5.5 dpc by immunohistochemistry. In *Egfp*-OE embryos, integrin β3 was strongly detected in cells surrounding the embryos (Figure 4E). Immunohistochemistry analysis of PRL3D1 using serial sections confirmed that integrin β3 was expressed in primary TGCs. In contrast, integrin β3 was restricted to a small portion of the cells in *Cdx2*-OE embryos (Figure 4E). PRL3D1 exhibited the localization similar to that of integrin β3 in serial sections, suggesting that integrin β3 expression was repressed by *Cdx2* overexpression and integrin β3 was only detected in a few primary TGCs produced by escaping from LV-*Cdx2* transduction. Taken together, these results indicate that *Cdx2* down-regulation is required for the mTE to acquire migration ability as well as to differentiate, and thus that *Cdx2* down-regulation promotes implantation progression.

## Discussion

In the present study, we performed RNA-seq analysis with the mTE of pre- and peri-implantation blastocysts to capture the characteristic changes of the mTE during implantation. Surprisingly, it was revealed that gene expression of the mTE changes stage-specifically and dramatically from E3.5 to E4.5. For example, expression of the genes related to several metabolic pathways changed during the period. These results suggest that the mTE alters the metabolism and the metabolic requirements in accordance with implantation progression. In addition, we revealed that while the genes for cell adhesion and migration were upregulated by E4.0, the genes for glycolysis were upregulated later at E4.5. It is possible that glycolysis is important for facilitating the invasion process during implantation because it has been thought that lactate efflux from the blastocyst promotes invasion of the TE cells and angiogenesis in the uterus, and reduces the local immune response at the implantation site by creating a low pH microenvironment around the embryos (Gardner, 2015). Such stage-specific, stepwise and dramatic gene expression change within a day might be important for the orderly progression of implantation. Our RNA-seq data captured changes in the expression of many other genes involved in various cellular functions, and we expect that these results will contribute to elucidation of the whole picture of the regulatory mechanisms of implantation.

The TE invading the uterus share some features with cancer cells in terms of invading and growing in tissues, creating a blood supply, and suppressing the maternal immune systems (Holtan et al., 2009; Costanzo et al., 2018). Thus, it has been thought that cancer metastasis is a biological process similar to blastocyst implantation. On the other hand, it is also known that the invasion of TE cells into the uterus is restrictive, whereas cancer cells immoderately expand in tissues (Holtan et al., 2009; Costanzo et al., 2018). Our RNA-seq data revealed that the mTE shows several cancer-like transcriptional characteristics such as downregulation of p53 signaling pathway-related genes including tumor suppressor *Trp53* and *Cdkn1a*, and upregulation of cell migration- and glycolysis-related genes during implantation. In contrast, although epithelial-mesenchymal transition (EMT) is known as a hallmark of cancer metastasis (Holtan et al., 2009; Jordan et al., 2011), the mTE did not exhibit the transcriptional changes of EMT regulators (Supplementary Figure S10A). Thus, our RNA-seq data might enable to refer to the similarities and differences of invasion processes between implantation and cancer metastasis.

Stemness of mouse TSCs is supported by the networks of various transcription factors (Hemberger et al., 2020). In particular, ESRRB and SOX2 is located at the top of the networks (Adachi et al., 2013). On the other hand, ESRRB and SOX2 are not expressed in the TE prior to E4.5 (Adachi et al., 2013; Okamura et al., 2019; Suzuki et al., 2022), indicating they are not associated with initiation of mTE differentiation. EOMES forms a transcriptional factor complex with ELF5, and this complex drives the expression of undifferentiation marker genes (Latos et al., 2015b). We revealed that the mTE maintained the expression of *Eomes* and *Elf5* between E3.5 and E4.5 whereas up-regulating the differentiation marker genes. These results indicated that downregulation of these genes was dispensable to initiate mTE differentiation. It is known that homozygous deletion of *Eomes* blocks TE differentiation under the induction of blastocyst outgrowth (Strumpf et al., 2005). Therefore, it is suggested that *Eomes* is required to induce differentiation rather than maintain the undifferentiated state of the TE. Thus, the expression patterns and functions of undifferentiation marker genes may differ between pTE and the mTE. Furthermore, we revealed that repression of the *Cdx2* down-regulation in the mTE by LV-*Cdx2* transduction hindered mTE differentiation. Therefore, we have demonstrated that the function of CDX2 during the peri-implantation stage is to maintain the undifferentiated state, just as it is in the post-implantation stage. We further propose the possibility that the transcription factor *Cdx2* is a key upstream regulator governing undifferentiation state of the mTE.

Since TE-specific *Cdx2* overexpression inhibited blastocyst outgrowth in our experiments, we thought that *Cdx2* also regulated the expression of genes related to TE migration. Actually, when we used the RNA-seq data to screen the candidate genes whose expression was increased by *Cdx2* down-regulation in the mTE, various genes related to cell adhesion and migration were included among the screened genes. In particular, *Slc6a14*, *Slc16a3*, *Itga7*, *Itgav* and *Itgb3* are known to regulate migration of TE cells. SLC6A14 is an Na^+^/Cl^−^-coupled transporter for neutral and cationic amino acids, and uptake of leucine from uterine fluid through SLC6A14 stimulates the onset of TE motility (Van Winkle et al., 2006; González et al., 2012). SLC16A3 is a proton-linked monocarboxylate transporter and lactate efflux from the blastocyst through SLC16A3 regulates many aspects of the implantation process such as the facilitation of TE invasion and induction of angiogenesis in decidua (Gardner, 2015). Integrins are a α-β-heterodimeric transmembrane receptors and binding of uterus-derived ECM to the TE via integrins activates TE adhesion and invasion (Wang and Armant, 2002; Rout et al., 2004; Chaen et al., 2012). Thus, these are all genes encoding transporter or receptor proteins that pass the implantation regulatory factors between the blastocyst and the uterus. Therefore, we propose that *Cdx2* regulates migration of the mTE by mediating the blastocyst–uterus interactions.

Integrins are well known to regulate blastocyst implantation because implantation depends on interactions between the TE and the endometrial ECM (Wang and Armant, 2002). It has been known that β1-class integrin (integrin α5β1) efficiently strengthens fibronectin-binding activity of the blastocyst, while subsequent TE migration primarily depends on β3-class integrins (integrin αVβ3 and αIIbβ3) (Rout et al., 2004). We then analyzed the expression of the genes encoding the integrin subunits in the mTE and found that *Itgav* and *Itgb3* were drastically up-regulated over E3.5–4.5, while *Itga5* and *Itgb1* were highly expressed throughout E3.5–4.5 (Supplementary Figure S10B). Moreover, expression of *Itgav* and *Itgb3* was repressed, although that of *Itga5* and *Itgb1* was hardly affected in *Cdx2*-OE blastocysts (data not shown). Therefore, it is suggested that *Cdx2* down-regulation is a trigger to activate the β3-class integrin signaling for initiation of TE migration, while blastocysts are capable of integrin α5β1-mediated adhesion without *Cdx2* down-regulation. We thought this is the reason outgrowth was delayed rather than inhibited, and attachment stage of the implantation normally progressed in *Cdx2*-OE blastocysts. Given that expression of *Itgav* and *Itgb3* are regulated by *Cdx2* in the mTE, it is suggested that *Cdx2* down-regulation is a trigger to activate the β3-class integrin signaling for initiation of TE migration.

We also identified *Cited2* as one of the candidate gene whose expression exhibits inverse correlation with the expression of *Cdx2* in the TE. *Cited2* is a transcriptional co-factor regulating mouse placental development. Remarkably, *Cited2* is required for trophoblast differentiation, specifically differentiation into TGCs, spongiotrophoblasts and glycogen cells (Withington et al., 2006). Moreover, *Cited2* regulates the subcellular localization of SLC16A3 in the trophoblast and vascularization in the fetal region of the placenta through PDGF signaling (Moreau et al., 2014). Therefore, it might be that *Cited2* expression induced by *Cdx2* down-regulation is important for mTE differentiation, enhancement of mTE cell invasion via lactate secretion and vascularization between the embryonic–maternal interface in the decidua.

Our outgrowth experiments indicated that *Cdx2* downregulation is required to activate cell migration ability in the mTE. In contrast, Chicago sky blue injection revealed that *Cdx2* overexpression had little effect on the implantation rate of the blastocysts. Therefore, it is indicated that blastocyst attachment and initiation of decidualization are not disturbed by *Cdx2* overexpression. In contrast, several abnormalities were shown in the implantation sites of *Cdx2*-OE blastocysts: excessive vascular-like structures, hemorrhage and abnormal location of the embryos. It is known that paracrine signals from the primary TGCs regulate decidualization (Bany and Cross, 2006). Therefore, a lack of TGCs might cause incomplete decidualization, resulting in the abnormal implantation of *Cdx2*-OE embryos. Collectively, it is suggested that *Cdx2* down-regulation in the mTE is dispensable for blastocyst attachment process and initiation of decidualization, however that is required for mTE migration and normal progression of implantation.

In summary, we demonstrated that *Cdx2* in the mTE at peri-implantation stage has a dual role as (i) a positive regulator of undifferentiated state maintenance and (ii) a suppressor of cell migration. Thus, we propose that *Cdx2* functions as a gatekeeper for the invasion process during implantation. In colorectal and breast cancers, *Cdx2* is known as a tumor suppressor that antagonizes cancer metastasis by inhibiting cell migration (Gross et al., 2008; Wang et al., 2020; Yu et al., 2021). Therefore, we also propose that regulation of cell migration is a common function between the cancers and the mTE.

## Data Availability

The datasets presented in this study can be found in online repositories. The names of the repository/repositories and accession number(s) can be found below: DDBJ Sequence Read Archive, and accession number is DRA013954.
