# Supplementary material for: Dynamic Changes of Gene Expression in Mouse Mural Trophectoderm Regulated by Cdx2 During Implantation
Source: Front Cell Dev Biol. 2022 Aug 16;10:945241. doi: 10.3389/fcell.2022.945241 (PMC9425295; doi:10.3389/fcell.2022.945241)
Supplement: Supplementary file 1 [file Table1.docx]

Supplementary Material

# Supplementary Figures

**
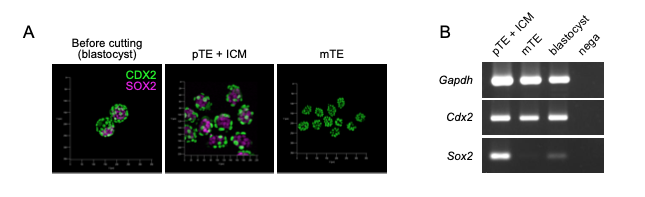
Supplementary Figure 1. Validation of the method used to isolate the mTE by a microblade.** (A) Immunostaining of CDX2 and SOX2 in E3.5 blastocysts (before cutting), isolated pTE and ICM, and mTE. (B) RT-PCR analysis of *Cdx2* and *Sox2*.

**
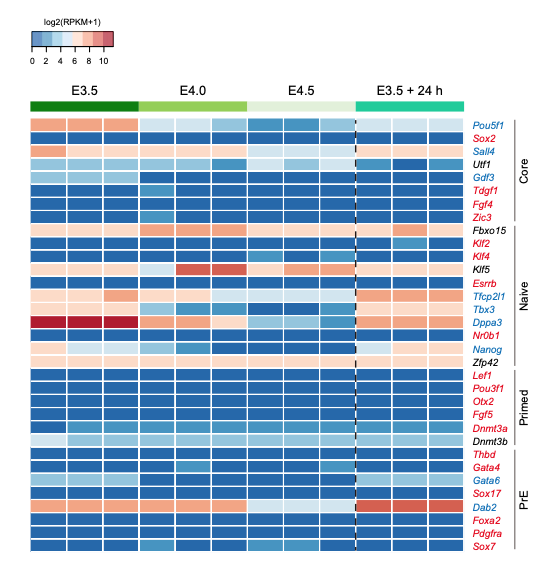
 Supplementary Figure 2. Expression patterns of epiblast and PrE marker genes in the mTE.** Heatmap showing the expression levels of epiblast and PrE marker genes in the mTE. The expression of the genes in red and blue were considerably low in all samples (RPKM < 10) and gradually reduced in vivo, respectively.


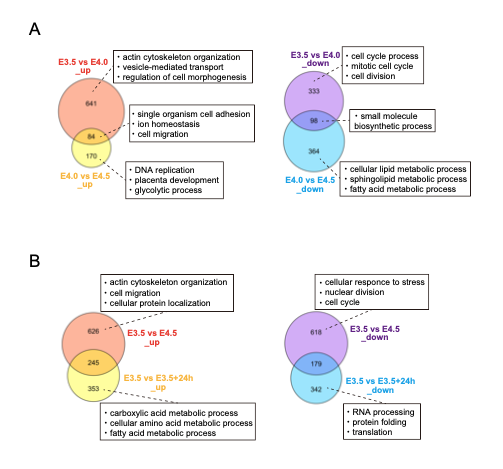


**Supplementary Figure 3. Common and specific DEGs between each stage.** (A) Venn diagram showing the overlap of genes up- and down-regulated between E3.5–4.0 and E4.0–4.5. Right columns of the Venn diagrams show the GO terms with preferential gene enrichment. (B) Venn diagram showing the overlap of genes up- and down-regulated between in vivo and in vitro. Right columns of the Venn diagrams show the GO terms with preferential gene enrichment.

**
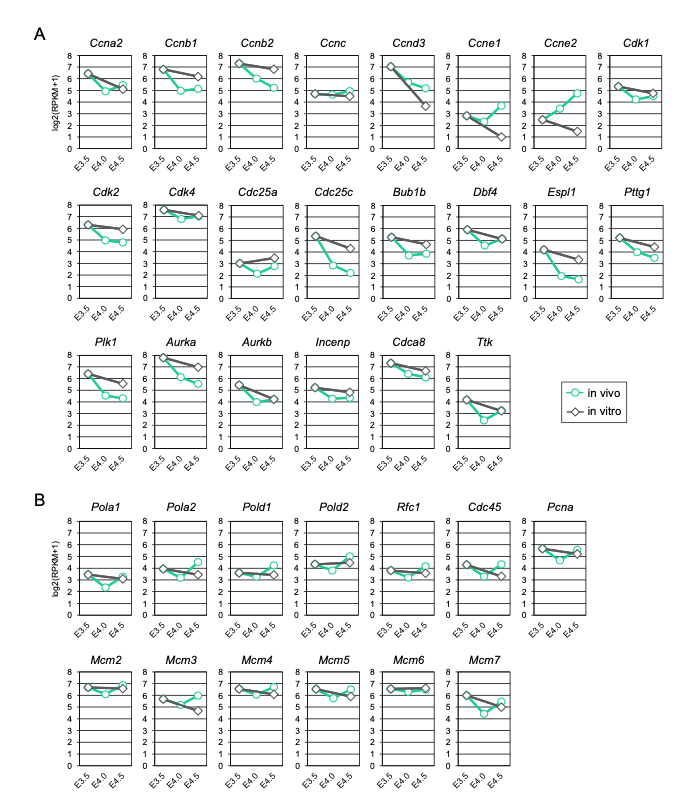
**

**Supplementary Figure 4. The mTE exhibited cell cycle arrest from E4.0.** (A) mRNA levels of cell cycle-related genes detected by RNA-seq analysis in mTE. (B) mRNA levels of DNA replication-related genes detected by RNA-seq analysis in mTE.

**
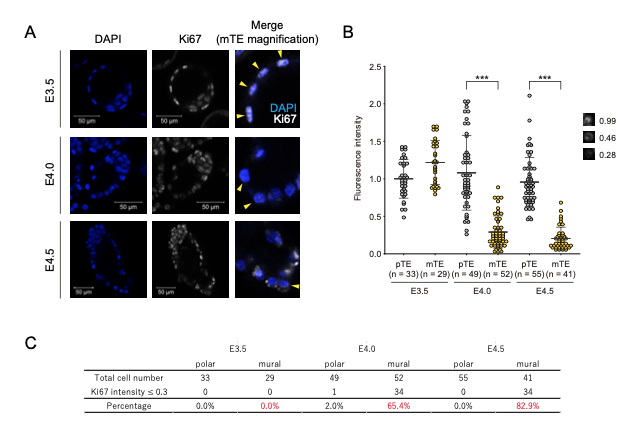
Supplementary Figure 5. Ki67 expression in the pTE and mTE of pre- and peri-implantation blastocysts.** (A) Immunostaining of Ki67 in blastocysts at E3.5, E4.0 and E4.5. Arrowheads indicate Ki67-positive mTE. Scale bar: 50 µm. (B) Comparison of Ki67 fluorescence intensity between pTE and mTE in E3.5, E4.0 and E4.5 blastocysts. Blastocysts were divided into three equal areas (polar, intermediate, mural) along the embryonic-abembryonic axis, and the fluorescence intensity in each nucleus containing in polar and mural areas was measured. Statistical significance was determined by Student’s *t*-test (****P* < 0.001) between polar and mural TE at each stage. n indicates the number of nuclei analyzed. Bars indicate mean ± S.D. (C) Percentage of Ki67-negative cells in pTE and mTE. Cells with Ki67 fluorescence intensity below 0.3 in (B) were defined as Ki67-negative cells.

**
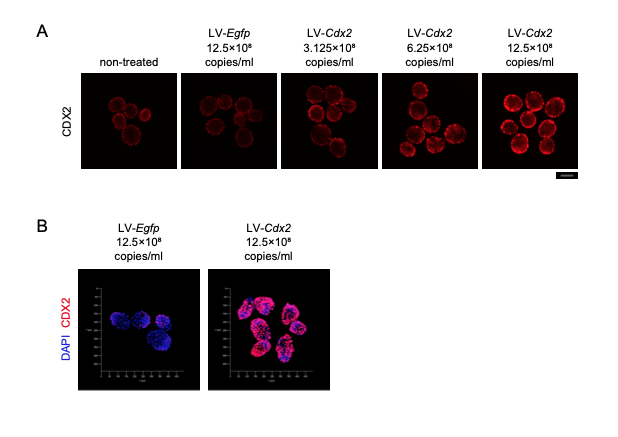
Supplementary Figure 6. *Cdx2*-OE blastocysts by LV-*Cdx2*-transduction.** Immunostaining of CDX2 in LV-*Egfp*- and LV-*Cdx2*-transduced blastocysts, cultured in vitro for 24 h after the transfection (A), and recovered from uteri about 40 h after blastocyst transfer (B). Scale bar in (A): 100 µm.

**
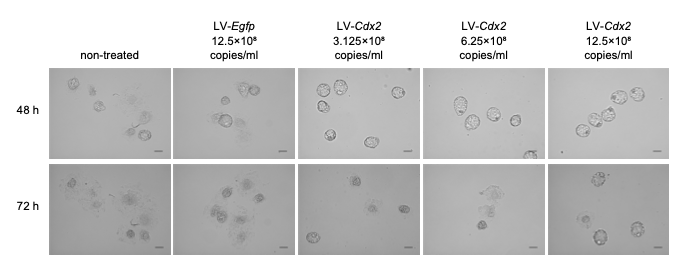
**

**Supplementary Figure 7. Outgrowth development of *Cdx2*-OE blastocysts.** Scale bars: 100 µm


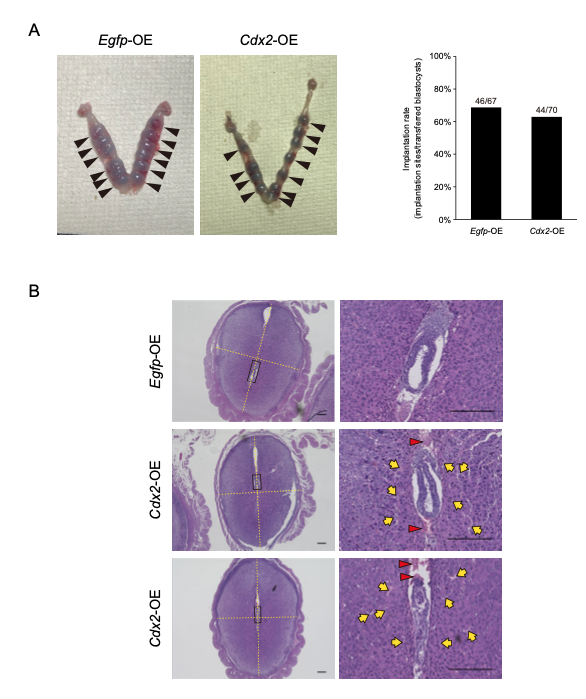


**Supplementary Figure 8.** *Cdx2*-OE embryos showed abnormal implantation. (A) Implantation sites detected by intravenous injection of Chicago sky blue dye (arrowheads). Implantation rates were calculated by implantation sites/transferred blastocysts. The numbers on top of the bars indicate the implantation sites/transferred blastocysts. (B) Uterus sections stained with H&E. Boxed regions in the left columns are shown at higher magnification in the right columns. Arrows and arrowheads indicate the vascular-like structures and hemorrhage in the implantation sites of LV-*Cdx2*-transduced embryos, respectively. Scale bar: 200 µm.

**
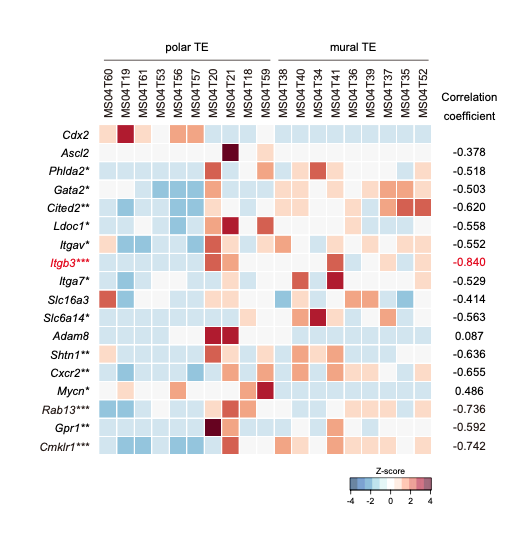
Supplementary Figure 9. Correlation between the expression of *Cdx2* and genes picked up in Fig. 4B.** Heatmap showing the expression levels of *Cdx2* and genes picked up in Fig. 4B for each cell of E4.5 pTE and mTE. These results were obtained by re-analyzing the published single-cell RNA-seq datasets of E4.5 blastocysts. Numerals indicate the Spearman’s correlation coefficient (**P* < 0.05, ***P* < 0.01, ****P* < 0.001).

**
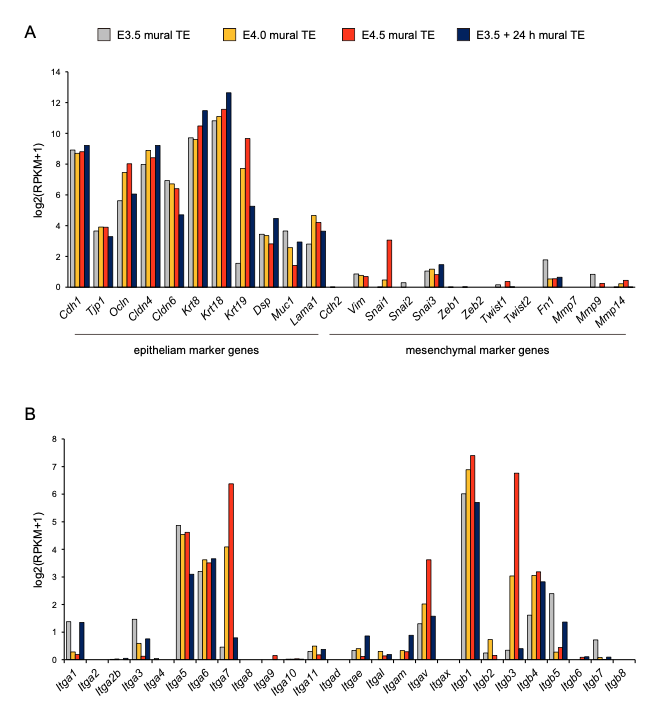
Supplementary Figure 10. mRNA levels of EMT marker genes and integrin subunit genes detected by RNA-seq analysis in the mTE.** (A) mRNA levels of EMT marker genes. (B) mRNA levels of integrin subunit genes.

# Supplementary Table1. Primer information for RT-qPCR.

Supplementary Table2. Antibodies used in the present study.
